# Alterations in common marmoset gut microbiome associated with duodenal strictures

**DOI:** 10.1038/s41598-022-09268-9

**Published:** 2022-03-28

**Authors:** Alexander Sheh, Stephen C. Artim, Monika A. Burns, Jose Arturo Molina-Mora, Mary Anne Lee, JoAnn Dzink-Fox, Sureshkumar Muthupalani, James G. Fox

**Affiliations:** 1 Division of Comparative Medicine, Massachusetts Institute of Technology, Cambridge, MA USA; 2 Centro de Investigación en Enfermedades Tropicales (CIET), Universidad de Costa Rica, San José, Costa Rica; 3 Department of Biological Sciences, Wellesley College, Wellesley, MA USA; 4 Present Address: Merck Research Laboratories, Merck, South San Francisco, CA USA

**Keywords:** Machine learning, Microbial communities, Gastroenterology, Gastrointestinal diseases, Gastrointestinal models

## Abstract

Chronic gastrointestinal (GI) diseases are the most common diseases in captive common marmosets (*Callithrix jacchus*). Despite standardized housing, diet and husbandry, a recently described gastrointestinal syndrome characterized by duodenal ulcers and strictures was observed in a subset of marmosets sourced from the New England Primate Research Center. As changes in the gut microbiome have been associated with GI diseases, the gut microbiome of 52 healthy, non-stricture marmosets (153 samples) were compared to the gut microbiome of 21 captive marmosets diagnosed with a duodenal ulcer/stricture (57 samples). No significant changes were observed using alpha diversity metrics, and while the community structure was significantly different when comparing beta diversity between healthy and stricture cases, the results were inconclusive due to differences observed in the dispersion of both datasets. Differences in the abundance of individual taxa using ANCOM, as stricture-associated dysbiosis was characterized by *Anaerobiospirillum* loss and *Clostridium perfringens* increases. To identify microbial and serum biomarkers that could help classify stricture cases, we developed models using machine learning algorithms (random forest, classification and regression trees, support vector machines and k-nearest neighbors) to classify microbiome, serum chemistry or complete blood count (CBC) data. Random forest (RF) models were the most accurate models and correctly classified strictures using either 9 ASVs (amplicon sequence variants), 4 serum chemistry tests or 6 CBC tests. Based on the RF model and ANCOM results, *C. perfringens* was identified as a potential causative agent associated with the development of strictures. *Clostridium perfringens* was also isolated by microbiological culture in 4 of 9 duodenum samples from marmosets with histologically confirmed strictures. Due to the enrichment of *C. perfringens *in situ, we analyzed frozen duodenal tissues using both 16S microbiome profiling and RNAseq. Microbiome analysis of the duodenal tissues of 29 marmosets from the MIT colony confirmed an increased abundance of *Clostridium* in stricture cases. Comparison of the duodenal gene expression from stricture and non-stricture marmosets found enrichment of genes associated with intestinal absorption, and lipid metabolism, localization, and transport in stricture cases. Using machine learning, we identified increased abundance of *C. perfringens*, as a potential causative agent of GI disease and intestinal strictures in marmosets.

## Introduction

In captive common marmosets, gastrointestinal (GI) diseases are the most common and widespread clinical finding^1,2^. Inflammatory bowel disease (IBD) prevalence is reported to be as high as 28–60% in captive marmosets and presents with diarrhea, weight loss, enteritis, muscle atrophy, alopecia, hypoproteinemia, anemia, elevated liver enzymes, failure to thrive and mortality^1,3^. In addition to IBD, a novel GI disease has been described in young adult to adult marmosets characterized by duodenal dilation or stricture near the major duodenal papilla^4–6^. Clinical signs, such as diarrhea, weight loss, or poor weight gain, resemble IBD but increased vomiting is also observed. This syndrome was associated with hypoalbuminemia, hypoglobulinemia, hypoproteinemia, hypocalcemia (total), elevated alkaline phosphatase, anemia, and in some cases, leukocytosis^5^. Histologically, duodenal mucosal ulcerations with associated chronic-active granulocytic and lympho-histiocytic inflammation were observed. Thus far only two institutions have reported this disease characterized by duodenal dilation amongst captive marmosets^6^. This duodenal syndrome was found in 21.9% of necropsy cases in a Japanese institution^4^. Within our institution, we observed a 13% prevalence of stricture cases during this 2 year study. However, 91.3% of cases (21 of 23 cases) within our colony were observed in marmosets sourced from the former New England Primate Resource Center, yielding a 26% prevalence when only considering marmosets from this source (MIT^NE^). In this study, we investigated the changes observed in the gut microbiome of MIT^NE^ marmosets that could be associated with the development of duodenal strictures.

The human GI tract harbors trillions of microorganisms from at least 400 species that compose the intestinal microbiota^7,8^. In healthy individuals, the microbiome influences many physiological functions such as extracting nutrients, maintaining the gut mucosal barrier, training immune cells and protecting against pathogens^9^. Dysbiosis occurs due to loss of beneficial microbes, expansion of pathobionts (opportunistic microbes), or reduction of microbial diversity. Dysbiosis has been associated with human diseases, including IBD, irritable bowel syndrome, obesity, psoriasis, rheumatoid arthritis, autism spectrum disorders, and *Clostridioles difficile* infection^9,10^. As the microbiome has been associated with human GI diseases, factors affecting the microbiome in non-human primates (NHP) are being explored, such as species, social structure, environment and diet^11–14^. Captivity and diets fed to captive marmosets have been associated with microbial diversity loss, shifts in the *Firmicutes*:*Bacteroidetes* ratio, and increased GI disease and mortality^11,12,14^. Dietary specialists, such as marmosets, are more susceptible to captivity-associated dietary changes^14^. Marmosets are exudivores that consume large amounts of indigestible oligosaccharides from tree gums^15^ and may harbor specific gut microbes dedicated to carbohydrate metabolism. Currently, few reports on the marmoset microbiome are available^16–21^. We recently published a longitudinal marmoset microbiome study examining both healthy marmosets and marmosets presenting clinically with IBD which surveyed 503 samples from 140 marmosets from four different sources^22^. Interestingly, even after months or years of husbandry at MIT, the most notable differences in the microbiome were based on the animals’ source, while marmoset age or sex had little to no effect in the microbiome^22^.

In this study, we compared the microbiome of 21 marmosets diagnosed with duodenal ulcer/strictures with the microbiome of 52 healthy, non-stricture marmosets imported from the same source [New England Primate Research Center (NEPRC)] and maintained at the MIT colony (MIT^NE^). “Stricture” animals were defined as marmosets that had or developed strictures based on clinical and histological assessments while “non-stricture” individuals were not clinically diagnosed with strictures and were not receiving chronic drug treatments during the study period. Healthy, non-stricture MIT^NE^ marmosets were included in our prior publication comparing the microbiome of MIT marmosets imported from different sources^22^. The current study evaluated fecal and rectal swab samples collected over a 2 year period during physical examinations or necropsies. Serum chemistry and complete blood count (CBC) samples from both healthy marmosets and marmosets diagnosed with duodenal ulcer/strictures from the MIT^NE^ colony were routinely collected once a year during physical examinations or during necropsies within the same 2 year period^23^. We identified changes in both microbial communities and blood parameters that may serve as marmoset biomarkers for strictures and propose that marmosets may be useful animal models to study *Clostridium*-driven GI disorders, such as duodenal strictures.

## Results

### Effects of Duodenal strictures on microbiome of MIT^NE^ marmosets

**Figure 1:**
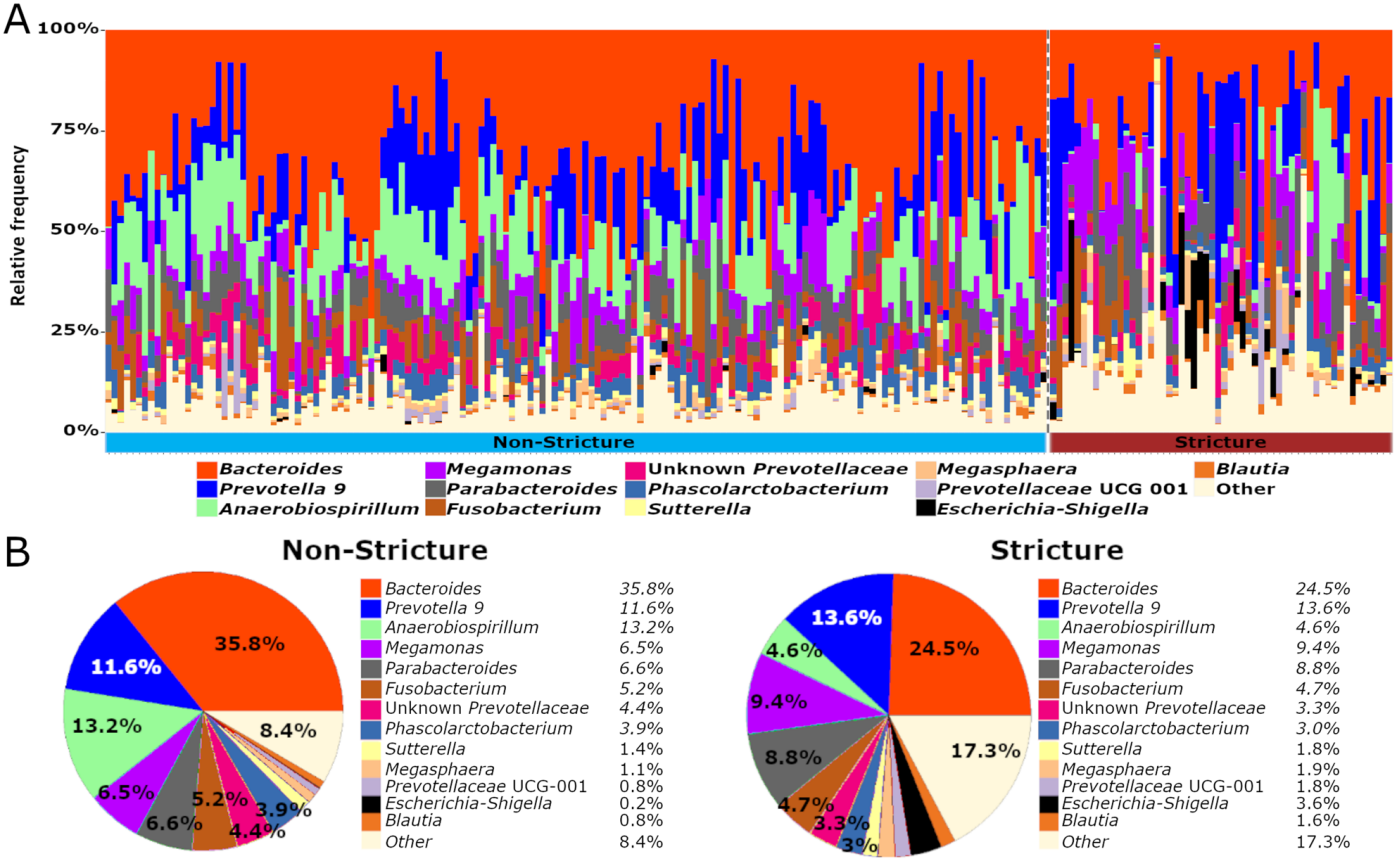
(**A**) Microbiome composition of individual samples at the genus level and (**B**) pie charts with average bacterial abundances of stricture (n = 57) and non-stricture marmosets (n = 153) show dysbiosis associated with stricture characterized by decreased *Bacteroides* and *Anaerobiospirillum* and increased *Megamonas*.

Of the 23 stricture cases identified at MIT during the study period, 21 marmosets belonged to the MIT^NE^ cohort, which had an estimated prevalence of 26% for this disease. This study focused on comparing biomarkers over a 2 year period collected from MIT^NE^ animals that were clinically or histologically diagnosed with strictures against healthy, non-stricture marmosets from the same source^22^. Marmosets included in this study were housed in a single building, kept isolated from marmosets from other sources for the duration of the 2 year study, and provided standardized diet, husbandry and veterinary care. In our analysis, the gut microbiome of healthy MIT^NE^ marmosets was characterized by high abundance of *Bacteroides*, followed by *Prevotella 9* and *Anaerobiospirillum*. We then compared the samples from the 52 non-stricture MIT^NE^ marmosets with samples from 21 MIT^NE^ marmosets diagnosed with strictures (Fig. 1, Table 1). The analysis identified 601 ASVs that could be collapsed into 128 genera with 47 genera accounting for 99% of total reads. Stricture marmosets had markedly different microbiomes compared to non-stricture animals. On average, a 32% decrease in *Bacteroides* was observed in stricture cases (35.8 ± 1.2% (mean ± standard error) abundance in non-stricture vs. 24.5 ± 2.0% in stricture). This decrease in *Bacteroides*, decreased the *Bacteroides*:*Prevotella 9* ratio from 3.1 in non-stricture to 1.4 in stricture. *Anaerobiospirillum*, the second most abundant genus in non-stricture marmosets (13.2 ± 0.7%), decreased to 4.6 ± 1.2% in stricture cases. The decreased abundance in these two genera was compensated by a 50% increase in *Megamonas* that was observed in stricture cases (6.5 ± 0.4% in non-stricture vs. 9.4 ± 1.0% in stricture) (Fig. 1). Analysis of Composition of Microbiomes (ANCOM), a log-ratio based statistical method that accounts for the compositional nature of microbiome data in differential abundance analysis between groups, was used to compare the two cohorts at the genus level. At this taxonomic level, ANCOM highlighted the decrease in *Anaerobiospirillum* observed in stricture cases and identified *Clostridium *sensu stricto* 1* as differentially expressed due to an increase in relative abundance observed in marmosets with stricture. Despite changes in microbial composition, no changes in alpha diversity were observed using multiple metrics (Chao1, Pielou’s Evenness, Observed OTUs and Shannon).

**Table 1:** Description of microbiome sample demographics of MIT^NE^.

	Unique non-stricture animals	Non-stricture samples	Unique stricture animals	Stricture samples
**Sex**				
Male	24	71	8	23
Female	28	82	13	34
Total	52	153	21	57
Age*				
2 and under	NA	66	NA	19
2–8	NA	70	NA	38
Over 8	NA	17	NA	0
Type**				
Rectal	51	100	12	21
Fecal	34	53	19	36

*Number of animals not reported as samples were collected over 2 year period and animals spanned multiple age groups.

**Fecal and Rectal Swabs were often collected from the same animal, so number of animals will be higher.

**Figure 2:**
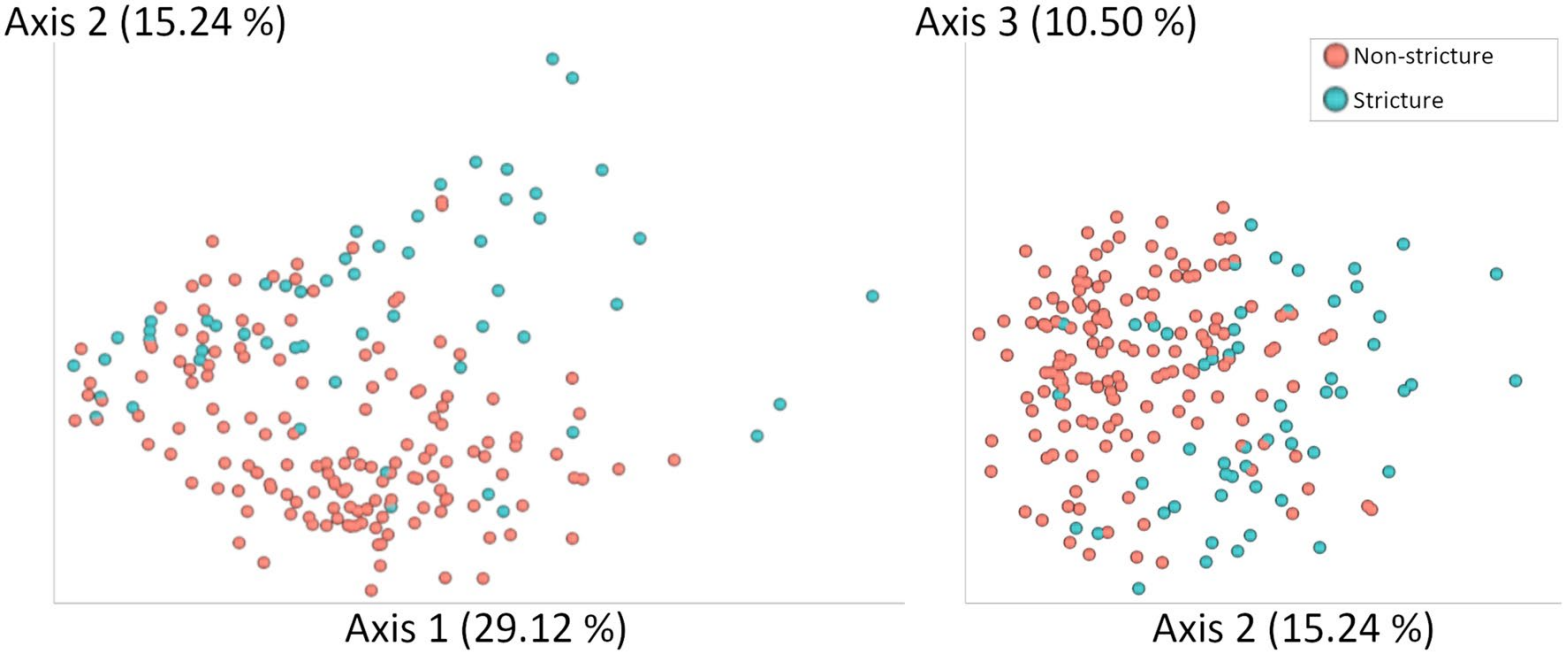
Weighted UniFrac PCoA plot depicting the top three principal components (PC) with (**A**) PC1 vs. PC2 and (**B**) PC2 vs. PC3. Clustering of microbiome profiles based on stricture status is observed with PC2 defining separation of healthy marmosets and stricture cases. However, beta-dispersion of both healthy and stricture cohorts is significantly different, implying differences in the variance observed in the two cohorts.

Using Principal Coordinate Analysis (PCoA) of Weighted UniFrac distances, 54.86% of variance was accounted for by 3 axes, with separation between stricture and healthy, non-stricture animals observed along the 2nd axis (Fig. 2). Using PERMANOVA (PERmutational Multivariate Analysis Of VAriance), a significant difference between the centroids of the healthy sample cluster and the stricture sample cluster was observed (PERMANOVA, *P* < 0.001 with F-statistic of 15.96), but beta-dispersion was also significantly different between clusters, implying that the spread of each cluster was different and could account for the difference observed between the centroids (beta-dispersion, *P* < 0.001) (Fig. 2).

### Identification of microbial and serum biomarkers of duodenal strictures using machine learning

**Figure 3:**
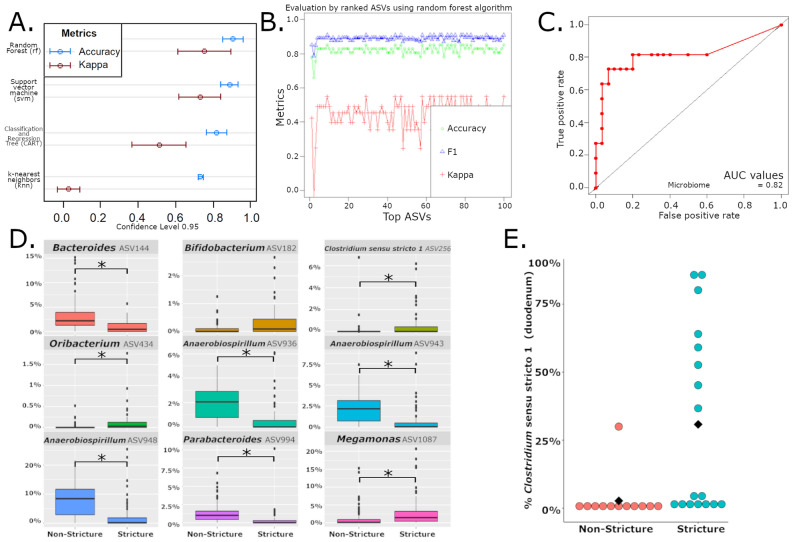
(**A**) Evaluation of 4 machine learning algorithms for classification of marmoset microbiomes based on stricture status. (**B**) Relationship between random forest model’s accuracy, F1 and Kappa on the number of ASVs used by the model to classify stricture and non-stricture samples with an 85% accuracy. (**C**) Area under the curve (AUC) of receiver operating characteristic (ROC) curves for random forest models using microbiome shows strong performance of models in classifying strictures and non-strictures. (**D**) Comparison of the relative abundance of the 9 ASVs identified in the random forest model in stricture and non-stricture samples. ANCOM analysis at the ASV level determined 8 of 9 ASVs to be significantly different between stricture and non-stricture. *= (W > 571) E) Evaluation of relative abundance of *Clostridium *sensu stricto* 1* reads in duodenal biopsies demonstrates increased abundance in stricture cases compared to non-stricture cases.

To identify potential microbial biomarkers associated with duodenal strictures, the microbiome data was analyzed using four machine learning algorithms (random forest (RF), support vector machines (SVM), classification and regression trees (CART) and k-nearest neighbor (KNN)) to determine the most accurate models for stricture classification based on marmoset microbiome profiles. For each algorithm, a subset of the dataset was used to train the model (training set) by providing the microbiome profile and the classification of the sample as a stricture or non-stricture sample. The remaining data was reserved to test the model’s accuracy (testing set). After the model was generated using the training set, the microbiome data from the testing set was provided to the model without the sample’s classifications. The model generated predictions based on the testing set microbiome profiles, which were then compared to the actual sample classifications in the testing set to determine the model’s accuracy. The model generation process was carried out iteratively to sample multiple subsets of the data and determine the robustness of the algorithm. Two metrics, accuracy and kappa, are shown for each model in Fig. 3a. Accuracy measures the percentage of correctly classified instances by comparing the clinically diagnosed ground truth data of the testing set with the model’s predictions. While kappa also compares the agreement of samples classified by the machine learning model with the ground truth data, it differs from accuracy by accounting the hypothetical probability of random agreements. Kappa values greater than 0.40 reflect moderate or substantial agreement between the model and the ground truth. Comparison of the four classification models shows that RF analysis provided the highest accuracy and kappa values when classifying microbiome profiles into “stricture” or “non-stricture.” Focusing on the RF model, we then evaluated the stability of three metrics (accuracy, kappa and F1 scores) to determine the least number of ASVs that maximized the three metrics. F1 score evaluates the model’s utility using both precision and recall (or sensitivity). The original analysis using QIIME2 generated a list of 601 ASVs. However, the RF model was able to detect the most important ASVs for classifying samples as “stricture” or “non-stricture.” Using a minimum of 4 ASVs, the three metrics in the RF model begin to stabilize, but we selected a 9 ASV model that presented the highest levels of accuracy, F1 and kappa observed with this model (Fig. 3b). The receiver operating characteristic (ROC) curve and area under the curve (AUC) value were calculated for the RF model using the 9 ASVs (Fig. 3c). The ROC curve had an AUC value of 0.82 with an accuracy of 85%, a sensitivity of 100% and a specificity of 45%, demonstrating a strong performance in classifying strictures and non-strictures. To better understand the effects of the 9 ASVs on the system, we identified the bacteria associated by QIIME2 with the ASVs and plotted the relative abundance of each based on their stricture status and determined that 8 of 9 ASVs were indeed significantly different by ANCOM analysis at the ASV level (Fig. 3d). Of these 9 ASVs, 3 *Anaerobiospirillum* ASVs, as well as *Bacteroides* and *Parabacteroides* ASVs, decreased in stricture cases. Increases were observed in ASVs from *Bifidobacterium, Clostridium *sensu stricto* 1, Oribacterium,* and *Megamonas*.

**Figure 4:**
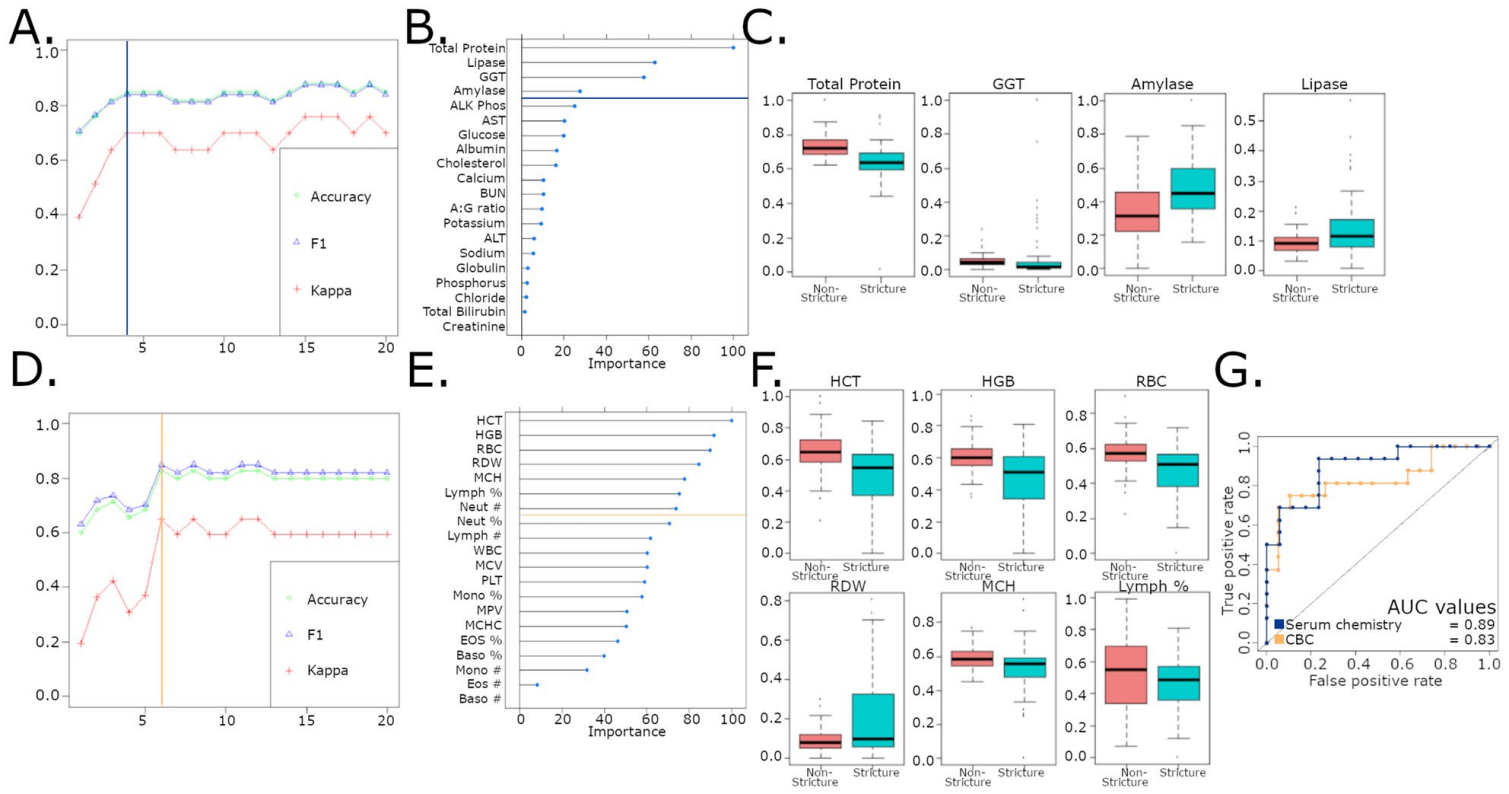
(**A**) Four serum chemistry parameters required for the optimal combination of accuracy and variable number in serum chemistry RF model. (**B**) Variables of importance for RF model using serum chemistry data to classify strictures. (**C**) Four most important parameters include Total Protein, GGT, amylase and lipase. Amylase and lipase are elevated in stricture cases suggesting pancreatic disease. (**D**) Six CBC parameters required for optimal combination of accuracy and variable number in CBC RF model. (**E**) Variables of importance for RF model using complete blood counts (CBC) to classify strictures. (**F**) 6 CBC parameters include HCT, HGB, RBC, RDW, MCH and lymphocyte %. 5 of 6 parameters indicate anemia. (**G**) Area under the curve (AUC) of receiver operating characteristic (ROC) curves for random forest models using serum chemistry (blue) or complete blood (yellow) count show strong performance in classifying strictures and non-strictures.

Next, we developed RF models using serum chemistry or CBC data to determine if “stricture” and “non-stricture” could be identified using blood analysis (Supp. Table 1). First, we evaluated the serum chemistry parameters needed to optimize accuracy, F1 and kappa, and determined that 4 serum chemistry parameters (total protein, lipase, gamma-glutamyl transferase (GGT) and amylase) classified “stricture” and “non-stricture” with 84.8% accuracy, a sensitivity of 76.5%, a specificity of 93.8% and AUC of 0.89 (Fig. 4a,b,g). Total protein and GGT decreased in stricture cases, while pancreatic markers, lipase and amylase, were increased in stricture animals (Fig. 4c). Using CBC data, the RF classifier identified 6 parameters (hematocrit (HCT), hemoglobin (HGB), red blood cell count (RBC), red cell distribution width (RDW), mean corpuscular hemoglobin (MCH) and lymphocyte percentage) that classified strictures with an accuracy of 82.8%, a sensitivity of 89.4%, a specificity of 75% and AUC of 0.83 (Fig. 4d–f). All variables, except RDW, decreased in strictures (Fig. 4g).

### Identification of *Clostridium* species based on sequencing reads

As ANCOM and the RF model highlighted the role of *Clostridium *sensu stricto* 1,* we further investigated the species that are encompassed by the *Clostridium *sensu stricto* 1* taxonomy, which included the following *Clostridium* species: *C. tetani, C. botulinum, C. kluyveri, C. acetobutylicum, C. novyi, C. perfringens* and *C. beijerinckii*. These species are generally considered pathogenic and indicative of less healthy and less diverse microbiota^24^. Using the representative sequences assigned to all *Clostridium *sensu stricto* 1* ASVs, we determined that 232,156 (69%) *Clostridium *sensu stricto* 1* reads shared > 99% identity over the 370 bp sequence with *C. perfringens*. Remaining reads matched with *C. baratii* (19%), *C. colicanis* (7%) and an unknown *Clostridium* species (6%). Importantly, ASV256, which increased sixfold in stricture samples, shared 100% identity with *C. perfringens*. We then sought to confirm the presence of *C. perfringens* by microbial culture followed by 16S rRNA Sanger sequencing of clinical isolates. *Clostridium perfringens* was isolated and confirmed by sequencing in 4 of 9 duodenum samples tested from marmosets with histologically confirmed strictures. The only other *Clostridium* isolate recovered from the 9 samples was identified as either *C. baratii* or *C. sardiniense*, a rare causative agent of botulism^25^.

### Increased *clostridium *sensu stricto* 1* abundance in the duodenum of stricture cases

As the microbiome analysis of the lower GI identified an increase of the *C. perfringens* ASV and we isolated the putative pathogen from duodenal tissue collected from the stricture site, we analyzed the microbiome using duodenal samples from stricture (n = 17) and non-stricture cases (n = 12). *Clostridium *sensu stricto* 1* was observed at greater than 1% abundance in 76% of strictures (13/17) but only in 16% of non-stricture cases (2/12). In 8 stricture cases, *Clostridium* was the most abundant genus with abundances ranging from 37 to 87%. Interestingly, one non-stricture sample with 30% abundance of *Clostridium *sensu stricto* 1* had duodenal pathology characterized by mild duodenal mucosal congestion (Fig. 3e).

### Effects of GI disease on gene expression of the small intestine

**Figure 5:**
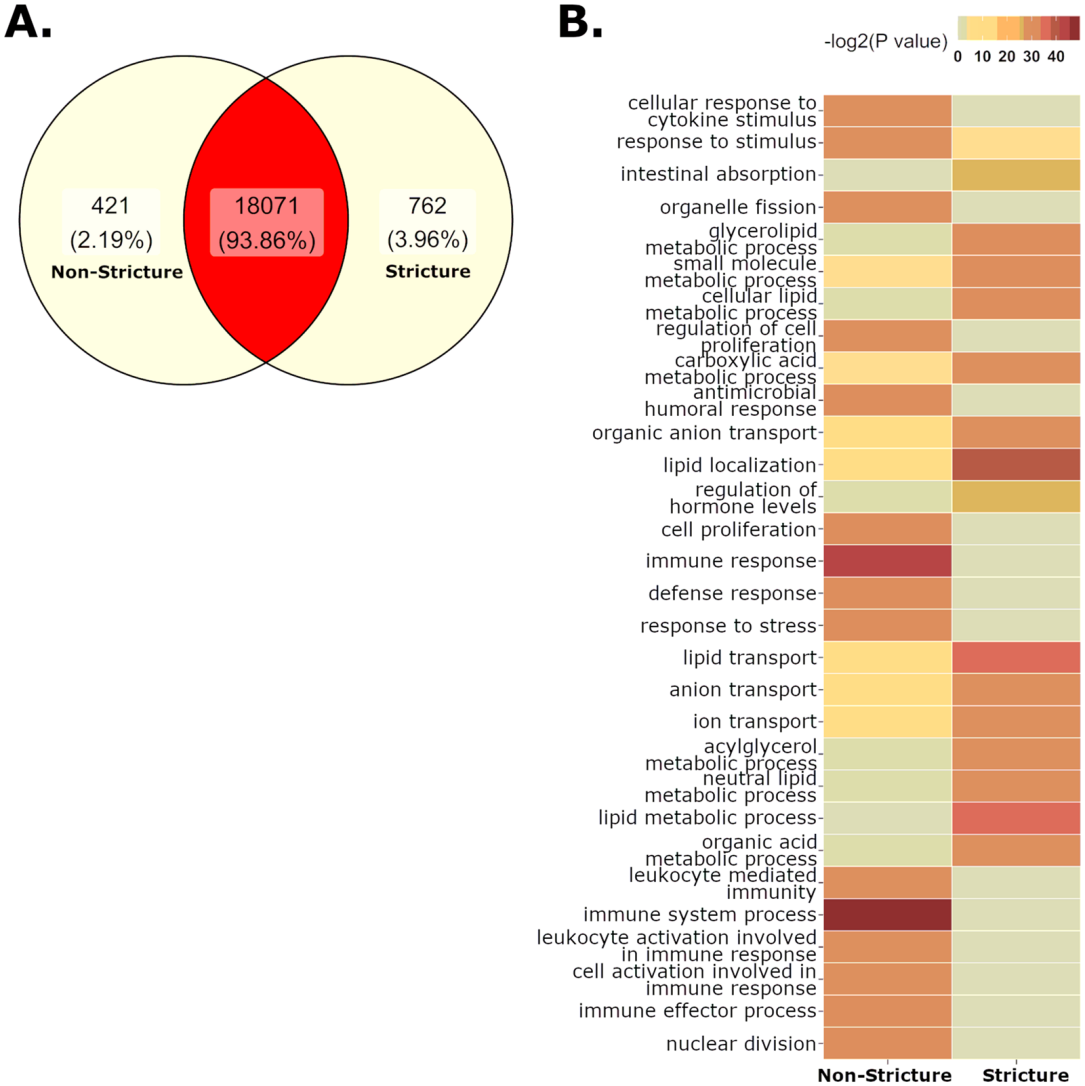
(**A**) Differentially expressed genes (DEG) (FDR < 0.05) in the duodenum of non-stricture and stricture cases. (**B**) Gene ontology (GO) sets enriched in stricture cases show upregulation of lipid metabolism, transport and localization. Non-stricture cases have enrichment of immune processes, possibly due to underlying pathology in necropsied animals with non-stricture diseases.

We tested whether strictures significantly altered marmoset transcriptomic profiles using RNA sequencing (RNAseq) on samples from non-stricture (n = 3) or stricture (n = 3) marmosets. Marmosets with strictures presented with gross thickening, duodenal stricture or ulceration (0.5–1 cm aboral to the major duodenal papilla). Duodenal tissue evaluated was immediately distal to the lesion (“stricture”) or in an equivalent anatomic region in IBD animals (“non-stricture”). However, IBD animals that served as non-stricture controls presented with thickened intestines that were grossly observed, and a diagnosis of duodenitis was noted. Comparing stricture and non-stricture duodenums, we identified 1,183 differentially expressed genes (DEG) (FDR < 0.05) (Fig. 5a, Supp. Table 2). To perform Gene ontology (GO) analysis, marmoset genes with official names were matched to *Homo sapiens* genes to retrieve Entrez IDs with associated GO categories. Analysis of this gene subset identified 903 DEGs with GO annotations. The top 15 biological processes (BP) with significant enrichment are listed in Table 2 (complete list Supp. Table 3). Stricture samples enriched BP sets involved with intestinal absorption, and lipid metabolism, localization, and transport (Fig. 5b, Supp. Fig. 1a). Stricture upregulated genes encompassed cholesterol-associated genes including apoliproteins (*APOB, APOA1* and *APOA4*), transport genes (*ABCG5, ABCG8, GRAMD1B,* and *STARD3*), metabolic genes (*DGAT1, CYP11A1,* and *CYP27A1*) and binding/absorption genes (*SOAT2, NPC1L1* and *SCARB1*) (Supp. Table 2). Other lipid-associated genes upregulated by stricture included genes associated with fatty acid binding proteins (*FABP1* and *FABP2*), peroxisomes (*PPARA, ABCD1, ACAA1* and *EPHX2*), ketogenesis (*HMGCS2*) and lipid synthesis (*GPAM, SREBF1, SCAP,* and *ACACB*). Enriched cellular membrane GO sets shared these lipid-associated genes due to functional overlap (Supp. Table 3, Supp. Fig.1). Interestingly, immunity-associated genes were more highly expressed in non-stricture duodenums (Fig. 5b, Supp. Fig. 2), possibly due to enteritis observed in IBD marmosets. These genes included antimicrobial responses (*LCN2*, *LYZ, MUC20*), toll-like receptors (*TLR2* and *TLR4*), superoxide-generating NADPH oxidase activity (*NOX1* and *DUOX2*), killer cell lectin-like receptor genes (*KLRB1, KLRC1, KLRD1,* and *KLRF1*), and chemokine activity and receptor binding (*CXCL1, CXCL10, TFF2* and *PF4*) (Supp. Table 2b). The transcriptional profile implies the activity of natural killer (NK) cells, neutrophils and MHC class I protein complex binding.

**Table 2:** Top gene ontology sets in stricture.

GO ID	Term	Ont	N	Up	Down	P. Up	P. Down
**Biological processes upregulated in the Duodenum in stricture**							
GO:0010876	Lipid localization	BP	292	14	44	1.08E−02	1.39E−12
GO:0006629	Lipid metabolic process	BP	1097	27	99	4.69E−01	1.02E−11
GO:0006869	Lipid transport	BP	262	13	39	1.05E−02	3.98E−11
GO:0046486	Glycerolipid metabolic process	BP	349	10	45	3.26E−01	1.70E−10
GO:0044281	Small molecule metabolic process	BP	1646	49	128	6.11E−02	1.80E−10
GO:0044255	Cellular lipid metabolic process	BP	842	22	79	3.65E−01	2.81E−10
GO:0006639	Acylglycerol metabolic process	BP	104	4	22	2.38E−01	1.00E−09
GO:0006638	Neutral lipid metabolic process	BP	105	4	22	0.24339141	1.22E−09
GO:0015711	Organic anion transport	BP	364	16	44	0.01443175	2.16E−09
GO:0006811	Ion transport	BP	1196	39	98	0.02879741	2.79E−09
GO:0006820	Anion transport	BP	459	19	50	0.01459	6.00E−09
GO:0006082	Organic acid metabolic process	BP	923	27	80	0.16158189	8.66E−09
GO:0019752	Carboxylic acid metabolic process	BP	845	27	75	0.07620236	9.78E−09
GO:0010817	Regulation of hormone levels	BP	377	10	43	0.41543649	1.90E−08
GO:0050892	Intestinal absorption	BP	35	0	12	1	2.04E−08
**Biological processes upregulated in the Duodenum in Non-Stricture**							
GO:0002376	Immune system process	BP	2192	110	86	1.24E−15	9.36E−01
GO:0006955	Immune response	BP	1482	83	50	4.21E−14	9.91E−01
GO:0019730	Antimicrobial humoral response	BP	49	13	0	7.97E−11	1.00E + 00
GO:0006950	Response to stress	BP	3001	122	106	9.11E−11	9.99E−01
GO:0008283	Cell proliferation	BP	1479	75	56	1.33E−10	9.35E−01
GO:0000280	Nuclear division	BP	322	30	7	1.81E−10	9.92E−01
GO:0002252	Immune effector process	BP	900	54	31	2.54E−10	9.58E−01
GO:0006952	Defense response	BP	1138	62	38	4.85E−10	9.84E−01
GO:0002443	Leukocyte mediated immunity	BP	598	41	21	1.05E−09	9.08E−01
GO:0048285	Organelle fission	BP	357	30	8	2.16E−09	9.92E−01
GO:0050896	Response to stimulus	BP	6351	203	305	3.03E−09	6.44E−02
GO:0042127	Regulation of cell proliferation	BP	1226	63	47	3.54E−09	9.01E−01
GO:0002366	Leukocyte activation involved in immune response	BP	559	38	21	5.77E−09	0.83699294
GO:0002263	Cell activation involved in immune response	BP	562	38	21	6.68E−09	0.84361261
GO:0071345	Cellular response to cytokine stimulus	BP	849	49	31	7.05E−09	0.91205517

## Discussion

GI diseases are the most prevalent clinical disease in captive common marmosets^1,2,26^, but the role of the microbiome is largely unknown. Recent literature demonstrates that housing in captive environments affects NHP microbiome composition, reduces alpha diversity, and alters host responses to disease^11,14,27^. In captivity, NHP microbiomes lose distinctive, wild microbiota and become dominated by *Prevotella* and *Bacteroides*, the most abundant genera in the modern human gut microbiome^8,11,28^. In the largest marmoset microbiome study to date, our previous report supported the hypothesis that captivity humanizes the primate microbiome, as *Bacteroides* and *Prevotella 9* were the most abundant genera with levels similar to those observed in human feces^8,22,28^. Within the MIT colony, NEPRC marmosets had the highest relative abundance of *Bacteroides* compared to the other marmoset sources^22^. MIT^NE^ marmosets had the highest *Bacteroidaceae* abundance (37%) and the lowest *Prevotellaceae* levels (17%), and were most susceptible to strictures, a novel GI disease in marmosets^4,5^. This duodenal syndrome was found in 21.9% of necropsy cases in a Japanese institution^4^, while MIT^NE^ marmosets had a 26% prevalence. Clinical signs include vomiting, bloating, weight loss and palpable thickening of the duodenum that can be visualized through radiography and ultrasound^4,5^. As strictures were most prevalent in the NEPRC-sourced colony, we compared microbiome samples from 21 NEPRC-sourced marmosets that developed strictures with samples from 52 non-stricture, NEPRC-sourced marmosets. While captivity increases susceptibility to GI disease in marmosets, the comparison of marmosets from a single-source and maintained within a single institution helps normalize the effects of stress and diet, which can affect the microbiome^29^. Stricture-associated dysbiosis featured shifts the relative abundance of *Bacteroides*, *Anaerobiospirillum* and *Megamonas* (Fig. 1), but commonly used analyses, such as alpha and beta diversity, showed no significant changes or inconclusive results, respectively.

In order to gain further insights into role of the microbiome in duodenal strictures, we used machine learning to identify ASVs of importance that could help generate testable hypotheses. Our analysis utilized four classifiers: RF, SVM, CART and KNN, which usually outperform traditional supervised classifiers^30–33^. Due to inherent differences in each algorithm, we benchmarked the methods to help identify the correct algorithm for classification of strictures and non-strictures given our dataset, and avoid the potential for bias and overfitting that exists when only a single algorithm is evaluated^33,34^. After evaluating the performance of the four methods, we found that random forest models had the best performance based on both accuracy and kappa metrics. In our RF model of the microbiome data, the model was optimized with 9 of the 601 ASVs generated in the QIIME2 workflow. Model stability observed with only a small portion of the data is desirable as selection of relevant features from noisy data, that is dimensionality reduction, is one of the main tasks in machine learning^33,35–37^. After reaching the optimal number of ASVs, the inclusion of other ASVs to the model adds redundant data, and eventually noisy data, without a significant improvement in model performance. As data from only 9 ASVs is required to correctly differentiate the stricture and non-stricture samples, these ASVs represent candidate biomarkers of the two states, which streamlines hypothesis generation and testing. Both our ANCOM analysis and RF model highlighted the importance of decreases in *Anaerobiospirillum* and increases in *Clostridium *sensu stricto* 1* in stricture cases. The consensus between two different analyses led us to further investigate *Anaerobiospirillum* and *Clostridium *sensu stricto* 1*. While *Anaerobiospirillum* has been previously reported in healthy marmosets, dogs, and cats^38,39^, these bacteria may cause GI disease in humans^38^. However, *Anaerobiospirillum* was present in high abundance in our healthy marmosets, and reduced levels were seen in stricture cases.

Using both microbial culture and sequence analysis, we determined that *C. perfringens* was observed at higher levels in the duodenal lesions of diseased animals. *C. perfringens* is a known GI pathogen that can encode multiple toxins (alpha, beta, epsilon, iota, perfringolysin O, and enterotoxin)^24^. In marmosets and other NHP, *C. perfringens* can cause gas gangrene and gastric dilatation syndrome^40–42^. Of note, *C. perfringens-*induced gas gangrene was reported in the Japanese vivarium that first reported duodenal strictures in captive marmosets^40^. In the United Kingdom, *C. perfringens* is one of the top 5 causes of foodborne death^43^, and has been linked to diarrhea, *Clostridial* necrotizing enteritis (CNE), necrotizing enterocolitis (NEC), ulcerative colitis (UC) and enterotoxemia in humans and other mammals^24,44^. CNE is a necrotizing inflammation of the small intestine that can induce mild diarrhea or severe abdominal pain, vomiting and ulcers^24^. NEC predominantly affects infants due to intestinal immaturity or dysbiosis^24,44^. While these symptoms match the clinical presentation of duodenal strictures in marmosets, they are non-specific. However, both small and large intestinal strictures developed in 11–29.5% of NEC infants and could occur up to 20 months post-NEC diagnosis^45,46^. Based on the site of *C. perfringens* infection at the junction of the duodenum and the common bile duct, we hypothesize that bile acid (BA) deregulation due to dysbiosis or antibiotic treatment may have facilitated *C. perfringens* infection. Antibiotic usage in infants has been linked with increased NEC risk^47^, and antibiotics are commonly prescribed to treat NHP GI diseases. Furthermore, *C. perfringens* was overrepresented in dogs with chronic enteropathy, an IBD-like disease, and bacterial abundance was regulated by secondary BAs (deoxycholic acid and lithocholic acid) that are produced by gut bacteria^48,49^. Due to its status as a known pathogen and presence in the site of injury, we propose that *C. perfringens* is a potential causative agent of duodenal disease in marmosets.

In addition to the role of *C. perfringens,* our serum chemistry and clinical chemistry-based RF models were highly sensitive in accurately classifying strictures. Decreased total protein levels are often observed with GI disease and may indicate poor digestion/absorption. The importance of amylase and lipase in our stricture model is supported by clinical findings of cholecystitis and secondary pancreatitis^5^. Secondary pancreatitis, attributed to extension from duodenal ulcers, was observed in 15 of 17 cases scored^5^. In the CBC-based model, HCT, HGB, RBC, RDW, and MCH relate to red blood cell function and suggested anemia. Anemia, a common finding in marmosets with strictures and IBD^3,5^, is also a risk factor for NEC in humans^50^. Interestingly, transcriptomic analysis of strictures showed enrichment of lipid metabolism and intestinal absorption genes, which may reflect enterocyte damage and is consistent with lipidomic alterations induced by *C. perfringens* alpha-toxin, a phospholipase C^51^. Increased expression of *FABP1* and *FABP2* was observed. These genes encode for liver and intestinal fatty-acid binding proteins (LFABP and IFABP), respectively, and are often used as biomarkers of GI diseases, including NEC^47^. To our knowledge, correlations of gut *FABP2* levels with serum IFABP levels have not been described, but we hypothesize that increased expression might be a compensatory mechanism triggered by enteritis. While increased inflammatory responses were not observed due to the lack of healthy control tissue, based on the *C. perfringens* infection, development of enteritis, anemia and strictures and deregulation of lipid metabolism, we believe marmosets could be developed as a model to investigate the mechanisms of bacterially-driven CNE/NEC.

A potential limitation of this study is potential imbalance in the dataset due to the scarcity of stricture samples relative to non-stricture samples. Imbalanced datasets are commonly encountered in machine learning applications as real-world classification problems, such as fraud detection, medical diagnosis, etc., are usually imbalanced^52^. While the 1:3 ratio of stricture to non-stricture samples is not generally considered an imbalanced dataset, the application of our benchmarking strategy to iteratively sample the dataset and assess performance metrics gives us confidence of the robust model performance. Another limitation of this study was the inability to ethically obtain age-matched duodenal samples from healthy, MIT^NE^ marmosets due to the early onset of the disease. In both analyses involving duodenal tissue, stricture samples were compared to duodenal tissue collected from sex-matched marmosets undergoing non-stricture-related necropsies. Additionally, we excluded non-stricture samples presenting with gross pathology due to other diseases, but we were not able to match the age and source. Previous microbiome studies have found minimal differences in the marmoset microbiome associated with age^18,22^. We have found that source strongly influence microbiome composition^22^, and while the microbiome may influence host responses, our interest in the RNAseq analysis was to elucidate the transcriptomic response to duodenal strictures. Future studies will focus on addressing small sample size concerns by banking MIT^NE^ duodenal tissues for further metagenomics and transcriptomic studies with MIT^NE^ stricture cases.

The common marmoset has emerged as useful NHP model for studying human disease as marmosets are small size, are easier to handle, are less costly to maintain than other NHP and mimic human disease^53^. Based on the presentation of bacterially-driven, intestinal inflammation in young adults presenting with no other illness, we propose that marmosets can be a viable model to investigate *C. perfringens*-associated enteritis. Better understanding of these disease profiles, the effects of diet and husbandry, and their inherent robustness to insults and disease will be helpful in promoting animal health, developing better models of human disease and understanding how to modulate microbial communities.

## Materials and methods

### Ethics statement

All research was conducted under an animal use protocol approved by the MIT Institutional Care and Use Committee (IACUC). The facility where this research was conducted is accredited by the AAALAC, International and adheres to principles stated in the Guide for the Care and Use of Laboratory Animals. Methods were carried out in accordance with the ARRIVE guidelines. Animals are cared for by a large staff of highly qualified veterinarians, veterinary technicians, and animal caretakers, who undergo substantial training to ensure only the highest quality animal care and use.

### Animals

Common marmosets (*Callithrix jacchus*) were housed at the Massachusetts Institute of Technology in Cambridge, MA, and were originally imported from the New England Primate Research Center (NEPRC) in 2014. This source is referred to as MIT^NE^. All animals were housed in pairs or family groups within one vivarium at MIT, an AAALAC International accredited facility. This study included 32 male and 41 female marmosets. Of the 52 healthy (non-progressor) animals, 28 were female and 24 were male, while in the stricture (progressor) cohort, 13 were female and 8 were male (Table 1). Samples from non-stricture marmosets were collected from marmosets ranging between 0.31 and 13.01 years of age, while samples from stricture animals were collected from marmosets aged 0.86–6.08 years of age. The animal holding room temperature was maintained at 74.0 + /−2°F with a relative humidity of 30–70%. The light cycle was maintained at a 12:12 h light:dark cycle. Marmosets were housed in cages composed of stainless-steel bars and polycarbonate perches with the following dimensions: 30″ W × 32″ D × 67″ H). Each cage had a nest box made of polycarbonate attached the outside of the cage. Other cage furniture present in the cages included hammocks, hanging toys, and manzanita wood branches. Foraging enrichment in the form of dried acacia gum-filled branches and forage board were provided weekly. Cages were spot-cleaned daily and removed for sanitization on a biweekly rotation.

All animals received a base chow diet of biscuits (Teklad New World Primate Diet 8794). Initially, biscuits were soaked in water for at least 20 min, but the practice was then changed to a pour-on/pour-off soak only. About halfway through the 2 year period encompassing this study, biscuit prep protocol reverted to the original practice of a 20 min soak to alleviate any concerns that soaking duration could be contributing to the development of duodenal ulcers. In addition to the base chow, a cafeteria-style supplemental offering of fruits (e.g. bananas, blueberries, mangoes, apples and grapes), vegetables (e.g. carrots, vegetable blend), acacia gum, and additional protein sources including hard-boiled eggs, mealworms, cottage cheese or ZuPreem (Premium Nutritional Products, Inc., Mission, KS).

On a semiannual basis, preventative health physical exams were performed on all colony animals. Rectal swabs and fecal samples were collected and screened for potentially pathogenic bacteria (including *Salmonella* spp., *Shigella* spp, beta-hemolytic *E.coli*, *Klebsiella* spp., and *Campylobacter* spp.) and parasites (including *Enterobius* spp., *Entamoeba* spp., *Giardia* spp., *Taenia* spp., and *Cryptosporidium* spp.). Additional fecal and rectal swab samples were collected between 2016 and 2018 for microbiome analysis. Intradermal testing for *Mycobacterium tuberculosis* was performed semiannually as well. All animals derived from progenitor stock were negative for squirrel monkey cytomegalovirus, *Saimiriine herpesvirus 1, Saimiriine herpesvirus 2, *and measles virus. Complete blood count and serum chemistry analysis were performed on an annual basis and during diagnostic workup of clinical cases. Hematology analysis was performed by the MIT DCM diagnostic laboratory using a HemaVet 950 veterinary hematology analyzer (Drew Scientific, Oxford, CT). Serum chemistry analysis was performed by Idexx Laboratories (Westbrook, ME). Serum chemistry and complete blood counts data were collected from the clinical records from the MIT colony. Investigators collecting samples were aware of health status, but investigators processing samples were blinded.

### Bacterial culture methods

Duodenal tissue and duodenal contents collected from MIT common marmosets during necropsies performed by clinical veterinarians and veterinary pathologists were evaluated. Representative sections of major organs were collected, fixed in 10% neutral buffered formalin, embedded in paraffin, sectioned at 5 µm, and stained using hematoxylin and eosin (HE) for scoring by a boarded veterinary pathologist. Samples were flash frozen in vials containing Brucella broth in 20% glycerol and frozen at − 80 °C. Samples from stricture and non-stricture cases were selected. The tissues were thawed in an anaerobic atmosphere (10% CO_2_, 10% H_2_, 80% N_2_), and were homogenized with freeze medium with tissue grinders. The homogenate was divided into the following aliquots. For aerobic culture, the homogenates were plated onto chocolate agar, blood agar, MacConkey agar, and Brucella Broth medium containing 10% FCS. The plates were incubated at 37 °C in 5% CO_2_ for 24–48 h. For anaerobic culture, the homogenates were plated onto pre-reduced Brucella Blood Agar plates (BBL) and inoculated into thioglycollate broth. The cultures were incubated at 37 °C in an anaerobic chamber (Coy Lab Products) with mixed gas (10% CO_2_, 10% H_2_, 80% N_2_) for 48 h. For microaerobic culture to detect the growth of *Helicobacter* spp., the homogenates were plated onto selective antibiotic-impregnated plates (50 μg/ml amphotericin B, 100 μg/ml vancomycin, 3.3 μg/ml polymyxin B, 200 μg/ml bacitracin, and 10.7 μg/ml nalidixic acid)^54^ and Brucella Blood Agar plates after passing through 0.65 µm syringe filter. The plates were placed into a vented jar filled with mixed gas (10% CO_2_, 10% H_2_, 80% N_2_) and incubated at 37 °C for up to 3 weeks. The plates were checked every 2–3 days for growth. Aliquots of the homogenates were also used for DNA extraction using the High Pure PCR Template Preparation kit (Roche Molecular Biochemicals). Bacterial DNA was then subjected to 16S rRNA gene sequence analysis using conserved primers 9F 5′-GAGTTTGATYCTGGCTCAG-3′ and 1541R 5′-AAGGAGGTGWTCCARCC-3′ creating a 1.5 kb product from the 16S rRNA gene^55^. The amplicons were purified with a QIAquick PCR Purification kit (Qiagen) and directly sequenced using an ABI Prism BigDye terminator cycle sequencing ready reaction kit on a genetic analyser 3500 (Applied Biosystems).

### 16S microbiome profiling

Fecal DNA was extracted using the DNeasy PowerLyzer PowerSoil Kit, and DNA was amplified using universal primers of F515 (GTGYCAGCMGCCGCGGTAA) and R926 (CCGYCAATTYMTTTRAGTTT) to target the V4 and V5 regions of bacterial 16S rRNA fused to Illumina adaptors and barcode sequences as described previously^56^. Individual samples were barcoded and pooled to construct the sequencing library, followed by sequencing with an Illumina MiSeq instrument to generate pair-ended 300 × 300 reads. Sequencing quality was inspected using FastQC^57^. Reads were processed using QIIME 2–2018.6 within the MicrobiomeHelper v. 2.3.0 virtual box^56,58^. Briefly, primer sequences were trimmed using the cutadapt plugin^59^. Forward and reverse reads were truncated at 243 and 195 bases, respectively, prior to stitching and denoising reads into amplicon sequence variants (ASV) using DADA2. Samples with fewer than 7500 reads were excluded. ASVs present in fewer than 3 samples and with less than 24 counts were also excluded. Taxonomic classification was assigned using the custom 16S V4/V5 region classifier based on the SILVA 132 database (SSU Ref NR 99)^60^. Following initial quality control, 601 ASVs proceeded to further analysis in the fecal microbiome analysis. Phylogenetic trees, composition, alpha rarefaction, beta diversity metrics and ANCOM (Analysis of Composition of Microbiome)^61^ were evaluated using built-in QIIME2 functions^62^. Microsoft Excel and R (v 3.6.3 at http://www.R-project.org/) were used to perform statistical analyses and graphically represent data. Duodenal tissue samples flash frozen in liquid nitrogen were obtained from necropsies performed by clinical veterinarians and veterinary pathologists on 29 marmosets. Duodenal stricture samples were obtained from 7 males and 10 females ranging from 1.71 to 8.44 years of age. Non-stricture duodenal samples were obtained from 7 males and 5 females ranging from 1.82 to 10.4 years of age. Necropsy samples were collected from MIT^NE^ (n = 18) and two additional MIT sources (n = 11). Tissues were processed as described above for fecal samples to determine the relative abundance of *Clostridium *sensu stricto* 1* in the duodenum.

Additionally, R libraries phyloseq^63^, ggplot2 (2.2.1)^64^, caret^65^, vegan^66^, pROC^67^, and gtools^68^ were used to model microbiome data. 6 samples (5 rectal swabs and 1 fecal sample) were excluded from microbiome analysis due to poor sampling characterized by low quantities of visible fecal matter and a microbiome dominated by a single species (e.g. *Helicobacter*) that was discordant from samples from the same individual^19^. We analyzed the *Bacteroides*/*Prevotella* abundance ratio by taking the ratio of the averaged *Bacteroides* abundance and the averaged *Prevotella* abundance.

### Machine learning

Machine learning analysis was performed following the approach of our previous work using a strategy to benchmark classifiers to the identify the most suitable method for the each particular dataset^33^. Data from the microbiome, serum chemistries and complete blood counts were utilized to train classifiers. To minimize the stress caused by handling and sampling, banked samples collected during physical examinations were utilized. As testing needs varied for each exam, paired blood and microbiome samples were not available at every time point. Due to this limitation, we generated three independent models for the microbiome, serum chemistries and CBC data.

Data was normalized using min–max normalization. The data was then split using a single partition method and the classifiers were trained on 80% of the samples (training) and the discovered signatures were used to predict the populations on the remaining 20% of samples (testing) using the four machine learning approaches: support vector machines (SVM), random forest (RF), K-nearest neighbor (KNN), and Classification and Regression Trees (CART). A R script using the function in the Caret package utilized default parameters for training with cross-validation. The variable importance metric was calculated using the varImp function, which associated a specific value for each parameter. To evaluate the contribution of each parameter, the script ranked the parameters and calculated the variable importance starting with the ranked parameters with the highest score. This process was processed iteratively adding ranked parameters and recalculating the metrics with each subsequent addition until all ranked genes were evaluated. Metrics included accuracy (correct classification percentage), kappa value (inter-rater classification agreement), sensitivity, specificity, precision, recall, prevalence, and F1 score (harmonic average of the precision and recall). Based on the contribution of each parameter, we selected a K value of top parameters based on the following criteria: (1) the stability of the metrics (priority for accuracy, kappa, and F1) when the increment of ranked genes was done, and (2) minimum number K of parameters as possible. After the selection of the K value, ROC (Receiver-operating characteristic) curve and AUC (Area under the curve) value were calculated for each algorithm.

### RNAseq

Tissues were collected from the duodenum from marmosets with either stricture or IBD during necropsies performed by clinical veterinarians and veterinary pathologists. The three stricture marmosets were female sourced from the MIT^NE^ and aged 1.9, 2.7, and 2.9 years old (average 2.5 ± 0.4). The stricture duodenal samples were distal of the site of stricture (n = 3) and exhibited chronic active duodenitis and duodenal ulcers. Given the early onset of this disease, it was not ethically justifiable to collect duodenal samples from healthy MIT^NE^ females. Non-stricture marmosets were selected from MIT^CL^ female marmosets, aged 6.5, 6.6, and 7.0 years old (average 6.7 ± 0.2), that were undergoing necropsy due to IBD. Non-stricture samples were collected from IBD animals from the same region of the duodenum, and presented with mild thickening based on gross observations (n = 3). Tissues were flash frozed in liquid nitrogen and stored at − 80 °C. RNA was extracted using TRIzol reagent according to manufacturer’s instructions (Thermo Fisher Scientific). Total RNA was shipped on dry ice to Arraystar, Inc. (Rockville, MD) for quality control, rRNA depletion and sequencing on an Illumina HiSeq4000. FASTA files and the NCBI RefSeq GTF files for *Callithrix jacchus* based on the March 2009 (WUBSC 3.2/calJac3) assembly were obtained from the UCSC Genome browser^69^. Raw sequencing reads were mapped to an index built from *C. jacchus* FASTA files using the buildindex function in Rsubread^70^. 29,575 feature counts were obtained via the featureCounts function from the bam files using annotated exons in the *C. jacchus* GTF files. Analysis was then performed using edgeR^71,72^. Lowly expressed exons were removed using a cutoff of 10 counts per million (CPM) and presence in at least 2 samples. Normalization was performed using the Trimmed Mean of M-values (TMM) method using the calcNormFactors function. Following removal of lowly expressed exons and normalization, a dataset with 19,254 feature counts was further analyzed. Multidimensional scaling (MDS) plots and heatmaps were used to evaluate grouping of biological samples. Data were fitted using the glmQLFit function that uses a generalized linear model (GLM) implementing a quasi-likelihood (QL) fitting method. Quasi-likelihood F-tests were performed to test for differential expression using the decideTestsDGE function in edgeR using the Benjamini–Hochberg correction and False Discovery Rate (FDR) adjusted P-values of 0.05. To retrieve Gene Ontology (GO) classifications, *C. jacchus* genes that matched *Homo sapiens* gene names were assigned both the *C. jacchus* and *Homo sapiens* Entrez IDs. GO analysis was performed using limma^73^, AnnotationDbi^74^, GO.db^75^, topGO^76^, mygene^77^ and org.Hs.eg.db.

Data were visualized using ggplot2, gplots, Rgraphviz^78^, colorspace^79^ and ggVennDiagram^80^.

## Supplementary Information

Supplementary Information 1.

Supplementary Information 2.

Supplementary Information 3.

## Data Availability

RNAseq data is available under NCBI GEO Accession Number GSE156839. Microbiome data is available under NCBI BioProject PRJNA659472.

## Code availability

R scripts utilized to analyze data and generate figures are available at https://github.com/sheh-dcm/cj_microbiome.

## References

[1] Ludlage E, Mansfield K (2003). Clinical care and diseases of the common marmoset (*Callithrix jacchus*). Comp. Med..

[2] David JM, Dick EJ, Hubbard GB (2009). Spontaneous pathology of the common marmoset (*Callithrix jacchus*) and tamarins (*Saguinus oedipus*, *Saguinus mystax*). J. Med. Primatol..

[3] Baxter VK, Shaw GC, Sotuyo NP (2013). Serum albumin and body weight as biomarkers for the antemortem identification of bone and gastrointestinal disease in the common marmoset. PLoS ONE.

[4] Mineshige T, Inoue T, Yasuda M, Yurimoto T, Kawai K, Sasaki E (2020). Novel gastrointestinal disease in common marmosets characterised by duodenal dilation: A clinical and pathological study. Sci. Rep..

[5] Artim SC, Sheh A, Burns MA, Fox JG, Muthupalani S (2019). Abstracts of scientific presentations 2019 AALAS national meeting: P139 A Syndrome of duodenal ulceration with strictures in a colony of common marmosets (*Callithrix jacchus*). J. Am. Assoc. Lab. Anim. Sci..

[6] Fitz C, Goodroe A, Wierenga L, Mejia A, Simmons H (2021). Clinical management of gastrointestinal disease in the common marmoset (*Callithrix jacchus*). ILAR J..

[7] Ley RE, Peterson DA, Gordon JI (2006). Ecological and evolutionary forces shaping microbial diversity in the human intestine. Cell.

[8] Huttenhower C, Gevers D, Knight R (2012). Structure, function and diversity of the healthy human microbiome. Nature.

[9] Rajilić-Stojanović M (2013). Function of the microbiota. Best Pract. Res. Clin. Gastroenterol..

[10] Durack J, Lynch SV (2019). The gut microbiome: Relationships with disease and opportunities for therapy. J. Exp. Med..

[11] Clayton JB, Vangay P, Huang H (2016). Captivity humanizes the primate microbiome. Proc. Natl. Acad. Sci. USA.

[12] Malukiewicz, J., Cartwright, R. A., Dergam, J. A., *et al.* the effects of host taxon, hybridization, and environment on the gut microbiome of callithrix marmosets. *bioRxiv*. Published online July 22, **2019**:708255. doi:10.1101/708255

[13] Hicks AL, Lee KJ, Couto-Rodriguez M (2018). Gut microbiomes of wild great apes fluctuate seasonally in response to diet. Nat. Commun..

[14] Frankel JS, Mallott EK, Hopper LM, Ross SR, Amato KR (2019). The effect of captivity on the primate gut microbiome varies with host dietary niche. Am. J. Primatol..

[15] Rylands AB, de Faria D, Rylands AB (1993). Habitats, Feeding Ecology, and Home Range Size in the Genus Callithrix. Marmosets and Tamarins: Systematics, Behaviour, and Ecology.

[16] Kap YS, Bus-Spoor C, van Driel N (2018). Targeted diet modification reduces multiple sclerosis–like disease in adult marmoset monkeys from an outbred colony. J. Immunol..

[17] Ross CN, Austad S, Brasky K (2017). The development of a specific pathogen free (SPF) barrier colony of marmosets (*Callithrix jacchus*) for aging research. Aging.

[18] Reveles KR, Patel S, Forney L, Ross CN (2019). Age-related changes in the marmoset gut microbiome. Am. J. Primatol..

[19] Artim SC, Sheh A, Burns MA, Fox JG (2019). Evaluating rectal swab collection method for gut microbiome analysis in the common marmoset (*Callithrix jacchus*). PLoS ONE.

[20] Kobayashi R, Nagaoka K, Nishimura N (2020). Comparison of the fecal microbiota of two monogastric herbivorous and five omnivorous mammals. Anim. Sci. J..

[21] Zhu L, Clayton JB, Suhr Van Haute MJ (2020). Sex bias in gut microbiome transmission in newly paired marmosets (*Callithrix jacchus*). mSystems.

[22] Sheh A, Artim S, Burns M (2022). Analysis of gut microbiome profiles in common marmosets (*Callithrix jacchus*) in health and intestinal disease. Sci. Rep..

[23] Kramer R, Sheh A, Toolan CH, Muthupalani S (2021). Factors affecting hematologic and serum biochemical parameters in healthy common marmosets (*Callithrix jacchus*). J. Am. Assoc. Lab. Anim. Sci..

[24] Uzal FA, Navarro MA, Li J, Freedman JC, Shrestha A, McClane BA (2018). Comparative pathogenesis of enteric clostridial infections in humans and animals. Anaerobe.

[25] Mazuet C, Legeay C, Sautereau J (2017). Characterization of clostridium Baratii type F strains responsible for an outbreak of botulism linked to beef meat consumption in France. PLoS Curr..

[26] Potkay S (1992). Diseases of the callitrichidae: A review. J. Med. Primatol..

[27] McKenzie VJ, Jin Song S, Delsuc F (2017). The effects of captivity on the mammalian gut microbiome society for integrative and comparative biology. Integr. Comp. Biol..

[28] Arumugam M, Raes J, Pelletier E (2011). Enterotypes of the human gut microbiome. Nature.

[29] David LA, Maurice CF, Carmody RN (2014). Diet rapidly and reproducibly alters the human gut microbiome. Nature.

[30] Thanh Noi P, Kappas M (2017). Comparison of random forest, k-nearest neighbor, and support vector machine classifiers for land cover classification using sentinel-2 imagery. Sensors.

[31] Park H, Shimamura T, Imoto S, Miyano S (2018). Adaptive networkprofiler for identifying cancer characteristic-specific gene regulatory networks. J. Comput. Biol..

[32] Leung RKK, Wang Y, Ma RCW (2013). Using a multi-staged strategy based on machine learning and mathematical modeling to predict genotype-phenotype risk patterns in diabetic kidney disease: A prospective case-control cohort analysis. BMC Nephrol..

[33] Molina Mora JA, Montero-Manso P, García-Batán R, Campos-Sánchez R, Fernández JV, García F (2021). A first perturbome of Pseudomonas aeruginosa: Identification of core genes related to multiple perturbations by a machine learning approach. Biosystems.

[34] Chitra, P. K.A., Alias Balamurugan, S. A. Benchmark evaluation of classification methods for single label learning with R. *2013 IEEE Int Conf Emerg Trends Comput Commun Nanotechnology, ICE-CCN 2013*. Published online: 746–752. 10.1109/ICE-CCN.2013.6528603 (2013)

[35] Aguilera A, Palma M, Mata-Toledo R (2013). Determination of significant features to precancerous cervical classification. AASRI Proc..

[36] Li Y, Wang N, Perkins EJ, Zhang C, Gong P (2010). Identification and optimization of classifier genes from multi-class earthworm microarray dataset. PLoS ONE.

[37] Bermingham ML, Pong-Wong R, Spiliopoulou A (2015). Application of high-dimensional feature selection: Evaluation for genomic prediction in man. Sci. Reports.

[38] Malnick H, Williams K, Phil-Ebosie J, Levy AS (1990). Description of a medium for isolating *Anaerobiospirillum* spp., a possible cause of zoonotic disease, from diarrheal feces and blood of humans and use of the medium in a survey of human, canine, and feline feces. J. Clin. Microbiol..

[39] Albert K, Rani A, Sela DA (2018). The comparative genomics of Bifidobacterium callitrichos reflects dietary carbohydrate utilization within the common marmoset gut. Microb. Genom..

[40] Yasuda M, Inoue T, Ueno M (2016). A case of nontraumatic gas gangrene in a common marmoset (*Callithrix jacchus*). J Vet Med Sci..

[41] Christie RJ, King RE (1981). Acute gastric dilatation and rupture in *Macaca arctoides* associated with *Clostridium perfringens*. J. Med. Primatol..

[42] Meier TR, Myers DD, Eaton KA, Ko MH, Hankenson FC (2007). Gangrenous *Clostridium perfringens* infection and subsequent wound management in a Rhesus Macaque (*Macaca mulatta*). J. Am. Assoc. Lab. Anim. Sci..

[43] Holland D, Thomson L, Mahmoudzadeh N, Khaled A (2020). Estimating deaths from foodborne disease in the UK for 11 key pathogens. BMJ Open Gastroenterol..

[44] De La Cochetière MF, Piloquet H, Des Robert C, Darmaun D, Galmiche JP, Rozé JC (2004). Early intestinal bacterial colonization and necrotizing enterocolitis in premature infants: The putative role of Clostridium. Pediatr. Res..

[45] Janik JS, Ein SH, Mancer K (1981). Intestinal stricture after necrotizing enterocolitis. J. Pediatr. Surg..

[46] Phad N, Trivedi A, Todd D, Lakkundi A (2014). Intestinal strictures post-necrotising enterocolitis: Clinical profile and risk factors. J. Neonatal Surg..

[47] Neu J, Pammi M (2018). Necrotizing enterocolitis: The intestinal microbiome, metabolome and inflammatory mediators. Semin. Fetal Neonatal Med..

[48] Ridlon JM, Kang DJ, Hylemon PB (2006). Bile salt biotransformations by human intestinal bacteria. J. Lipid Res..

[49] Wang S, Martins R, Sullivan MC (2019). Diet-induced remission in chronic enteropathy is associated with altered microbial community structure and synthesis of secondary bile acids. Microbiome.

[50] Patel RM, Knezevic A, Shenvi N (2016). Association of red blood cell transfusion, anemia, and necrotizing enterocolitis in very low-birth-weight infants. JAMA J. Am. Med. Assoc..

[51] Manni M, Valero JG (2017). Lipidomic profile of GM95 cell death induced by *Clostridium perfringens* alpha toxin. Chem. Phys. Lipids.

[52] Tanha J, Abdi Y, Samadi N, Razzaghi N, Asadpour M (2020). Boosting methods for multi-class imbalanced data classification: An experimental review. J. Big Data..

[53] Carrion R, Patterson JL (2012). An animal model that reflects human disease: the common marmoset (*Callithrix jacchus*). Curr. Opin Virol..

[54] Fox JG, Dangler CA, Taylor NS, King A, Koh TJ, Wang TC (1999). High-salt diet induces gastric epithelial hyperplasia and parietal cell loss, and enhances helicobacter pylori colonization in C57BL/6 mice|cancer research. Cancer Res..

[55] Shen Z, Feng Y, Sheh A (2015). Isolation and characterization of a novel Helicobacter species, *Helicobacter jaachi* sp. Nov., from common marmosets (*Callithrix jaachus*). J. Med. Microbiol..

[56] Comeau AM, Douglas GM, Langille MGI (2017). Microbiome helper: A custom and streamlined workflow for microbiome research. mSystems.

[57] Andrews, S., Fast, Q. C. A quality control tool for high throughput sequence data. Published 2010. Accessed August 4, (2020). http://www.bioinformatics.babraham.ac.uk/projects/fastqc/

[58] Bolyen E, Rideout JR, Dillon MR (2019). Reproducible, interactive, scalable and extensible microbiome data science using QIIME 2. Nat. Biotechnol..

[59] Martin M (2011). Cutadapt removes adapter sequences from high-throughput sequencing reads. EMBnet J..

[60] Yilmaz P, Wegener Parfrey L, Yarza P (2014). SILVA and “All-species Living Tree Project (LTP)” taxonomic frameworks: Nucleic acids research Oxford academic. Nucleic Acids Res..

[61] Mandal S, Van Treuren W, White RA, Eggesbø M, Knight R, Peddada SD (2015). Analysis of composition of microbiomes: A novel method for studying microbial composition. Microb. Ecol. Heal. Dis..

[62] Lozupone C, Hamady M, Knight R (2006). UniFrac–an online tool for comparing microbial community diversity in a phylogenetic context. BMC Bioinform..

[63] McMurdie PJ, Holmes S (2013). phyloseq: An R package for reproducible interactive analysis and graphics of microbiome census data. PLoS ONE.

[64] Wickham H (2009). ggplot2: Elegant Graphics for Data Analysis.

[65] Kuhn M (2008). Building predictive models in R using the caret package. J. Stat. Softw..

[66] Oksanen J, Kindt R, Legendre P, et al. The vegan Package. Published online 2008. Accessed August 4, (2020). http://cran.r-project.org/

[67] Robin X, Turck N, Hainard A (2011). pROC: An open-source package for R and S+ to analyze and compare ROC curves. BMC Bioinform..

[68] Warns Gregory, Bolker Ben, L.T.. gtools: Various R Programming tools. Published online (2015).

[69] Lee CM, Barber GP, Casper J (2020). UCSC genome browser enters 20th year. Nucleic Acids Res..

[70] Liao Y, Smyth GK, Shi W (2019). The R package Rsubread is easier, faster, cheaper and better for alignment and quantification of RNA sequencing reads. Nucleic Acids Res..

[71] Robinson MD, Mccarthy DJ, Smyth GK (2010). edgeR: a Bioconductor package for differential expression analysis of digital gene expression data. Bioinformatics.

[72] Mccarthy DJ, Chen Y, Smyth GK (2012). Differential expression analysis of multifactor RNA-Seq experiments with respect to biological variation. Nucleic Acids Res..

[73] Ritchie ME, Phipson B, Wu D (2015). limma powers differential expression analyses for RNA-sequencing and microarray studies. Nucleic Acids Res..

[74] Pagès, H., Carlson, M., Falcon, S., Li, N. AnnotationDbi: Manipulation of SQLite-based annotations in Bioconductor. R package. Published online (2019).

[75] Carlson, M. GO.db: A set of annotation maps describing the entire Gene Ontology. R package. Published online (2019).

[76] Alexa, A., Rahnenfuhrer, J. topGO: Enrichment analysis for gene ontology. R package. Published online (2019).

[77] Mark, A., Thompson, R., Afrasiabi, C., Wu, C. mygene: Access MyGene.Info_services. R package version. Published online (2019).

[78] Hansen, K. D., Gentry, J., Long, L., *et al.* Rgraphviz: Provides plotting capabilities for R graph objects. R package. Published online. (2019).

[79] Zeileis, A., Fisher, J. C., Hornik, K., *et al.* colorspace: A Toolbox for Manipulating and Assessing Colors and Palettes. Published online March 14, 2019. Accessed August 4, (2020). http://arxiv.org/abs/1903.06490

[80] Gao, C.-H. ggVennDiagram: A “ggplot” Implement of Venn Diagram. R package. Published online (2019). https://cran.r-project.org/package=ggVennDiagram

