# Supplementary material for: Alterations in common marmoset gut microbiome associated with duodenal strictures
Source: Sci Rep. 2022 Mar 28;12:5277. doi: 10.1038/s41598-022-09268-9 (PMC8960757; doi:10.1038/s41598-022-09268-9)

Supp. Fig 1

A. Network of Biological Processes enriched in the duodenum of stricture cases

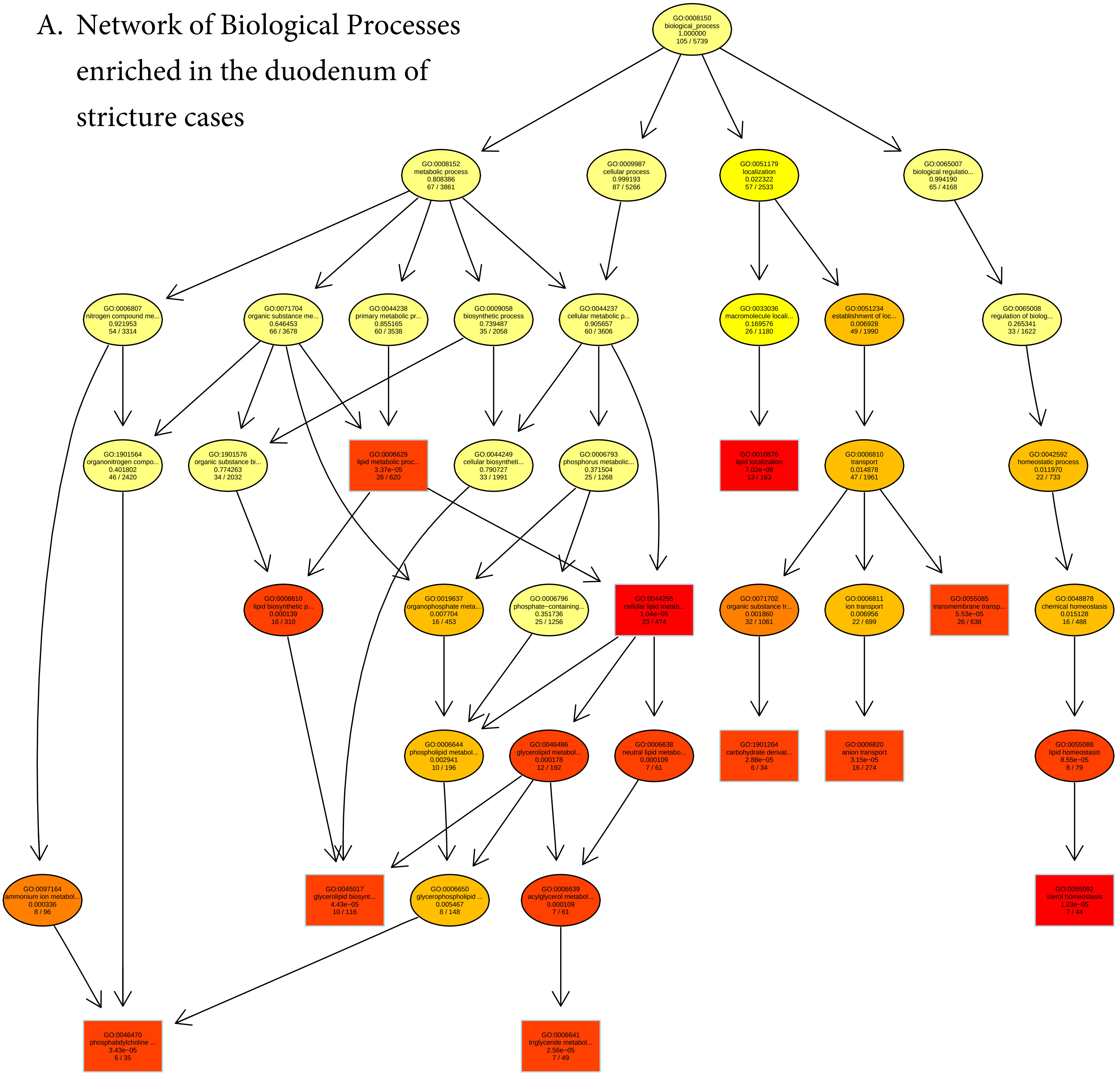

B. Network of Cellular Components enriched in the duodenum of stricture cases

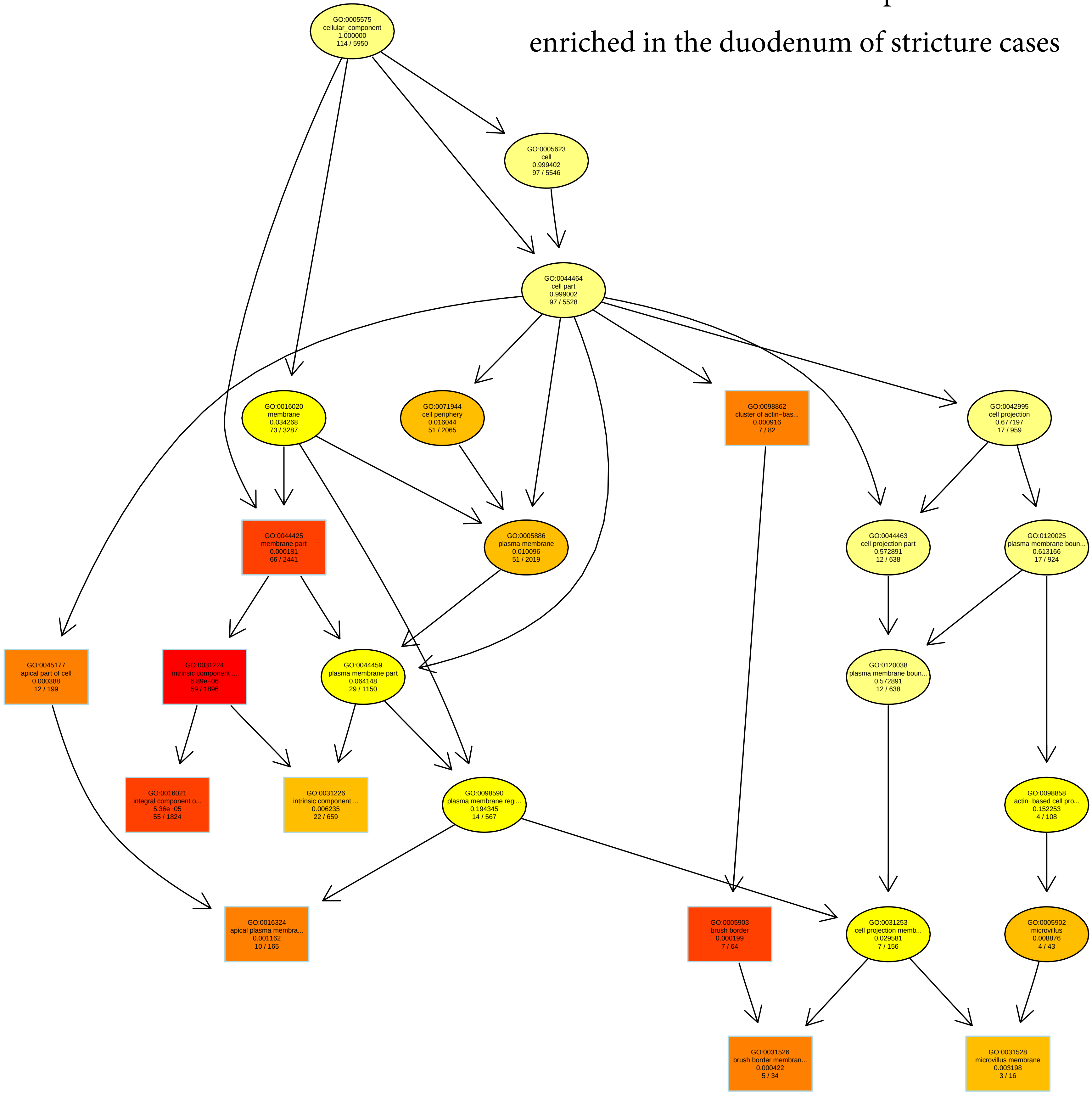

Supplement: Supplementary file 1 — Supplementary Information 1. [file 41598_2022_9268_MOESM1_ESM.pdf]
