# Supplementary figures and images for: Alterations in common marmoset gut microbiome associated with duodenal strictures
Source: Sci Rep. 2022 Mar 28;12:5277. doi: 10.1038/s41598-022-09268-9 (PMC8960757; doi:10.1038/s41598-022-09268-9)

Supp. Fig 2 Network of Biological Processes enriched in the duodenum of non-stricture cases

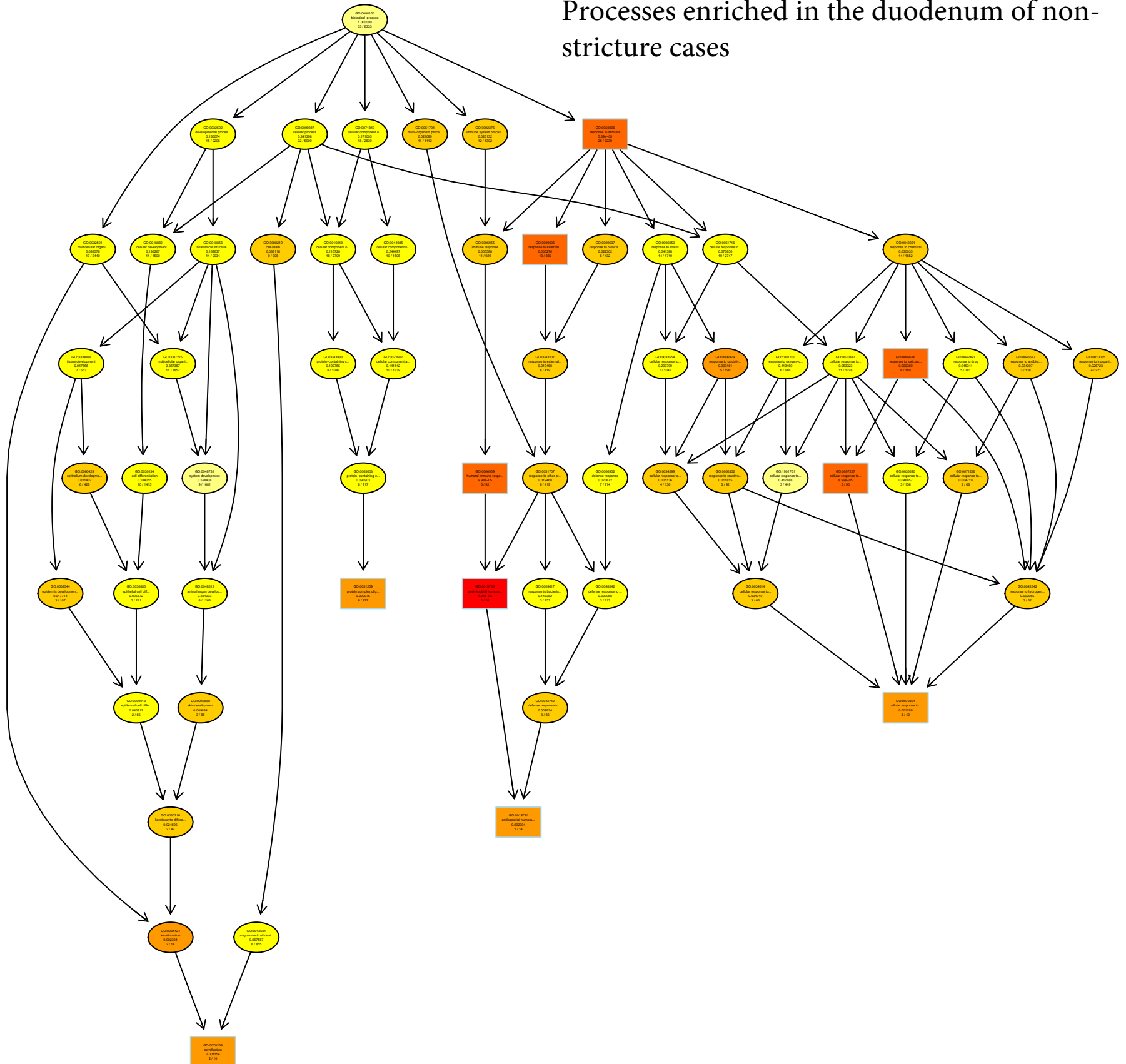

Supplement: Supplementary file 2 — Supplementary Information 2. [file 41598_2022_9268_MOESM2_ESM.pdf]
